# Baroreflex dynamics during the rest to exercise transient in acute normobaric hypoxia in humans

**DOI:** 10.1007/s00421-024-05485-4

**Published:** 2024-04-24

**Authors:** Anna Taboni, Nazzareno Fagoni, Timothée Fontolliet, Giovanni Vinetti, Guido Ferretti

**Affiliations:** 1 Department of Molecular and Translational Medicine, University of Brescia, Viale Europa 11, Brescia, Italy; 2 Institute of Mountain Emergency Medicine, Eurac Research, Bolzano, Italy; 3 Department of Anaesthesiology, Pharmacology, Intensive Care, and Emergencies, University of Geneva, Geneva, Switzerland

**Keywords:** Arterial baroreflex, Humans, Sequence method, Vagal withdrawal

## Abstract

**Purpose:**

We hypothesised that during a rest-to-exercise transient in hypoxia (H), compared to normoxia (N), (i) the initial baroreflex sensitivity (BRS) decrease would be slower and (ii) the fast heart rate (HR) and cardiac output (CO) response would have smaller amplitude (A_1_) due to lower vagal activity in H than N.

**Methods:**

Ten participants performed three rest-to-50 W exercise transients on a cycle-ergometer in N (ambient air) and three in H (inspired fraction of O_2_ = 0.11). R-to-R interval (RRi, by electrocardiography) and blood pressure profile (by photo-plethysmography) were recorded non-invasively. Analysis of the latter provided mean arterial pressure (MAP) and stroke volume (SV). CO = HR·SV. BRS was calculated by modified sequence method.

**Results:**

Upon exercise onset in N, MAP fell to a minimum (MAPmin) then recovered. BRS decreased immediately from 14.7 ± 3.6 at rest to 7.0 ± 3.0 ms mmHg^−1^ at 50 W (p < 0.01). The first BRS sequence detected at 50 W was 8.9 ± 4.8 ms mmHg^−1^ (p < 0.05 vs. rest). In H, MAP showed several oscillations until reaching a new steady state. BRS decreased rapidly from 10.6 ± 2.8 at rest to 2.9 ± 1.5 ms mmHg^−1^ at 50 W (p < 0.01), as the first BRS sequence at 50 W was 5.8 ± 2.6 ms mmHg^−1^ (p < 0.01 vs. rest). CO-A_1_ was 2.96 ± 1.51 and 2.31 ± 0.94 l min^−1^ in N and H, respectively (p = 0.06). HR-A_1_ was 7.7 ± 4.6 and 7.1 ± 5.9 min^−1^ in N and H, respectively (p = 0.81).

**Conclusion:**

The immediate BRS decrease in H, coupled with similar rapid HR and CO responses, is compatible with a withdrawal of residual vagal activity in H associated with increased sympathetic drive.

**Supplementary Information:**

The online version contains supplementary material available at 10.1007/s00421-024-05485-4.

## Introduction

In acute hypoxia, the control of heart rate (HR) and blood pressure is challenged by several mechanisms. To sustain the oxygen delivery, the decrease in blood oxygen concentration must be accompanied by an increase in cardiac output (CO), both at rest and during dynamic exercise (Stenberg et al. 1966; Ferretti et al. 1990; Adami et al. 2014). This CO increase is mainly sustained by an increase of HR with preserved stroke volume (SV) (Hartley et al. 1973; Siebenmann and Lundby 2015). Data suggest that these changes are the result of a different equilibrium in the autonomic nervous system, implying higher sympathetic and lower parasympathetic activity (Moore et al. 1986; Saito et al. 1988; Rowell et al. 1989; Sagawa et al. 1997; Boushel et al. 2001; Weisbrod et al. 2001; Halliwill and Minson 2002; Hopkins et al. 2003; Buchheit et al. 2004; Ferretti et al. 2005; Lador et al. 2008; Siebenmann and Lundby 2015).

When analysing the rest-to-exercise transient in normoxic condition, the first rapid HR response can be suppressed by vagal blockade with atropine (Fagraeus and Linnarsson 1976) suggesting a prompt withdrawal of the vagal activity (Fagraeus and Linnarsson 1976; Lador et al. 2006; Fontolliet et al. 2021). Following this concept, it has been hypothesised that conditions of reduced vagal activity at rest, as acute hypoxia, might be characterised by a reduced amplitude of the initial HR and CO response to exercise onset (phase I) (Lador et al. 2006). To the best of our knowledge, this hypothesis has been tested experimentally only once (Lador et al. 2008), although with a small number of observations (5 subjects). They reported that in acute hypoxia (inspired fraction of O_2_, F_I_O_2_, = 0.11) phase I of the cardiovascular response to exercise onset was characterised by smaller HR and CO amplitudes.

Hypoxia affects also arterial baroreflexes. Changes in slope of a baroreflex curve around the operating point (OP) (Kent et al. 1972) under different physiological conditions have been ascribed to modulation by the autonomic nervous system (Ogoh et al. 2005). The acute exposure to simulated high altitude (~ 4300 m) modifies the cardiac response of the carotid arterial baroreflex by resetting the entire response curve towards higher HR values and by decreasing the maximum gain of the reflex (Sagawa et al. 1997, neck pressure-suction technique). Coherently, application of the sequence method in acute hypoxia revealed a decrease of the spontaneous baroreflex sensitivity (BRS) around the OP (Klemenc and Golja 2011; Fisher et al. 2022).

Bringard et al. (2017), who applied the sequence method during the exercise transient, demonstrated an immediate reduction of the BRS at exercise start, which they ascribed to sudden vagal withdrawal, in line with the interpretation of the rapid cardiovascular response to exercise (Lador et al. 2006). Thus, if the vagal withdrawal conjecture holds, we would expect that, in acute normobaric hypoxia, a rest-to-light exercise transient would be characterised by: i) a smaller decrease of BRS and ii) a smaller amplitude of the phase I of HR and CO (corroborating the preliminary results of Lador et al 2008), than in normoxic conditions. The aim of the present study was to test these hypotheses by analysing the dynamics of arterial baroreflexes (Bringard et al. 2017; Taboni et al. 2021a, b, 2022) and the HR an CO kinetics (Lador et al. 2006; Fontolliet et al. 2021) during a rest-to-50 W exercise transient in normoxia and acute normobaric hypoxia.

## Materials and methods

### Subjects

Ten healthy subjects were enlisted (9 males and 1 female). Age, height, and body mass were 32 ± 6 years, 176 ± 9 cm, and 71 ± 13 kg, respectively. All subjects were moderately active. Their maximal aerobic capacity measured on the cycle ergometer (Lode Corival, Lode B.V., Groningen, The Netherlands) was 276 ± 67 W, 3.9 ± 1.0 W kg^−1^. They were already familiar with the laboratory protocols since they had participated in previous experiments. None reported history of cardiovascular, pulmonary, or neurological diseases or was taking medications at the time of the study. The subjects were asked to refrain from drinking coffee or smoking for 24 h before the experiments. All subjects gave their informed consent after having received a detailed description of the methods and experimental procedures of the study. Every subject was aware of the right of withdrawing from the study at any time without jeopardy. This study was performed in line with the principles of the Declaration of Helsinki. Approval was granted by the Commission Cantonale d’Éthique de la Recherche, Canton de Genève, CH (Date 11th July 2018–No. 2018–00913).

## Experimental procedure

The subjects came to the laboratory on one occasion, at least 2 h after a light meal. In the laboratory, ambient temperature was set to 24 ± 1 °C and barometric pressure was 731 ± 8 mmHg. After instrumentation and wearing cycling shoes, the subject took place on an electromagnetically braked cycle ergometer (Lode Corival, Lode B.V., Groningen, The Netherlands). The experimental protocol was carried out in normoxia (N) and in hypoxia (H), which were administered in random order and were separated by 30 min to allow for rest and hydration. Both in N and H, participants wore an oro-nasal mask (7450 V2 Mask™, Hans Rudolph, Inc., Shawnee, KS, USA) connected to an ultrasonic flowmeter (Spiroson®, ECO MEDICS AG, Duernten, Switzerland). In H, the experiments were carried out while the subject was breathing a hypoxic gas mixture (F_I_O_2_ = 0.11), which was delivered by means of a low resistance, two-way non-rebreathing T-shape valve (Hans Rudolph, Inc., Shawnee, KS, USA). The inhalation port was connected to a 200 l Douglas bag, used as pressure buffer system, and filled with a gas mixture containing 11% oxygen in nitrogen coming from a high-pressure tank. After connection, 10 min of quiet breathing were allowed to attain alveolar gas equilibration before performing the procedure.

The experimental protocol was as follows. After subjects’ instrumentation and equipment calibration, the subjects spent at least 10 min in quiet rest. Data were continuously recorded during the last 5 min of this period. At the 4th minute of resting recording, a 20 µl capillary blood sample was taken from the right earlobe for blood lactate concentration ([La]) measurement. At the same time, a 35–55 µl arterialised capillary blood sample was taken from the left earlobe for measurement of pH, of oxygen and carbon dioxide partial pressures (pO_2_ and pCO_2_, respectively), and of bicarbonate concentration ([HCO_3_^−^]). Afterwards, the subject performed three square-wave rest-to-exercise transients at the constant power of 50 W. The first exercise bout lasted 10 min to ensure at least 5 min of steady state condition; blood samples for [La], pH, pO_2_, pCO_2_, and [HCO_3_^−^] were taken at the 9th minute. After the first exercise bout, the subject rested on the cycle-ergometer of 6 min and then performed two additional 50 W exercise bouts lasting 5 min, separated by 6 min of rest. Both in N and H, every rest-to-exercise transient started without previous flywheel acceleration; this usually implies that the mechanical power necessary for the flywheel acceleration may compensate for the delayed activation of the magnetic brake of the cycle ergometer (Hibi et al. 1996).

### Measurements

Continuous non-invasive arterial blood pressure profiles were recorded at the medium finger (Portapres, Finapres® Medical Systems, Enschede, The Netherlands) and peripheral blood oxygen saturation (SpO_2_) was continuously monitored at the index finger of the left arm (Nellcor N-595, Medtronic, Minneapolis, MN, USA). The left arm was positioned on a support at the heart level. Beat-by-beat HR was recorded by electrocardiography (ECG100C module, BIOPAC® Systems Inc., Goleta, CA, USA). All signals were collected and sampled at 400 Hz (MP150 system with AcqKnowledge acquisition and analysis software, BIOPAC® Systems Inc., Goleta, CA, USA) and stored on a personal computer for subsequent analysis. [La] was measured by an enzymatic-amperometric method (Biosen C-Line Glucose and Lactate analyser, EKF Diagnostics, Cardiff, UK) on 20 μl capillary blood samples. Arterialised blood pH, pO_2_, pCO_2_, and [HCO_3_^−^] were measured (ABL800 FLEX, Radiometer, Brønshøj, Denmark) on 35–55 μl capillary blood samples.

### Data treatment

Arterial blood pressure profiles were analysed to obtain beat-by-beat values of systolic (SAP), diastolic (DAP), and mean (MAP) arterial pressure using the Beatscope® software (Finapres® Medical Systems, Enschede, The Netherlands). The same software provided a beat-by-beat calculation of SV by the Modelflow method (Wesseling et al. 1993). Beat-by-beat CO was calculated as the SV times the corresponding HR and total peripheral resistances (TPR) as the ratio between MAP and CO. Data in steady state conditions were computed on the last 5 min of the first resting period and of the 10 min exercise bout.

In steady state condition, at rest and at exercise, the BRS was calculated with the sequence method (Bertinieri et al. 1988), using MAP and R-to-R interval (RRi) as independent and dependent variable, respectively (Taboni et al. 2018). A phase shift of one beat between MAP and RRi was introduced (Steptoe and Vogele 1990), then, sequences of 3 or more consecutive beats characterised by consensual increase or decrease in MAP and RRi were identified. Within each sequence, the relationship between RRi and MAP was analysed by linear regression to compute the slope and the coefficient of determination (R^2^). When R^2^ > 0.85, the slope was retained (Iellamo et al. 1997). In steady state conditions, the mean slope of the RRi versus MAP relationship was considered representative of the BRS for each subject and the mean RRi and MAP value was considered as the corresponding OP.

BRS during the exercise transients was computed with the same approach as previously proposed for exercise onset (Bringard et al. 2017), breath holding onset (Taboni et al. 2021b), fast whole body tilting (Taboni et al. 2021a), and light-to-moderate exercise transient (Taboni et al. 2022). A phase shift of one beat for HR was introduced and the same criteria used at steady state were applied to retain sequences. The mean value of the RRi versus MAP relationship over the three repetitions of exercise transient was considered representative of the mean BRS for each subject. In N during the exercise transient, MAP showed an abrupt fall, until a minimum MAP value was recorded, then a recovery. Consequently, the baroreflex sequences retrieved during the first seconds of exercise in N have been grouped in two categories: (i) before the attainment of minimum MAP (“pre MAPmin” in Fig. 3), and (ii) the first sequence after the attainment of minimum MAP (“post MAPmin” in Fig. 3). In H during the exercise transient, MAP did not show any abrupt fall, which prevened identification of a minimum MAP. Thus, the baroreflex sequences retrieved during the first seconds of exercise in N have been grouped as follows: (i) the very first sequence identified after the exercise start (“first slope” in Fig. 3), and (ii) the second sequence identified after the exercise start ("second slope” in Fig. 3).

The dynamics of the CO and HR changes over time (*f*_(t)_) during the two exercise transients was analysed using a bi exponential model (Barstow and Molé 1987; Lador et al. 2006):

$$f_{{\left( t \right)}} = b + A_{1} \left( {1 - e^{{\frac{{ - t}}{{\tau _{1} }}}} } \right) + H_{{\left( {t - d} \right)}} A_{2} \left[ {1 - e^{{\frac{{ - (t - d)}}{{\tau _{2} }}}} } \right]$$

1 $$H_{{\left( {t - d} \right)}} = \left\{ {\begin{array}{*{20}c} {0, t - d < 0} \\ {1, t - d \ge 0} \\ \end{array} } \right.$$

where *b* is the baseline value, A is the response amplitude, *d* is the time delay, and τ is the time constant. The subscripts 1 and 2 refer to the initial (phase I) and the primary (phase II) components, respectively. *H*_(t−d)_ is the Heaviside function, when t < d it equals 0 and the last term of the right-hand branch of Eq. 1 cancels out. When the amplitude of one phase resulted equal to 0 l min^−1^ for CO or 0 bpm for HR, the corresponding time constant was not considered for the statistical analysis.

### Statistical analysis

**Fig. 1:**
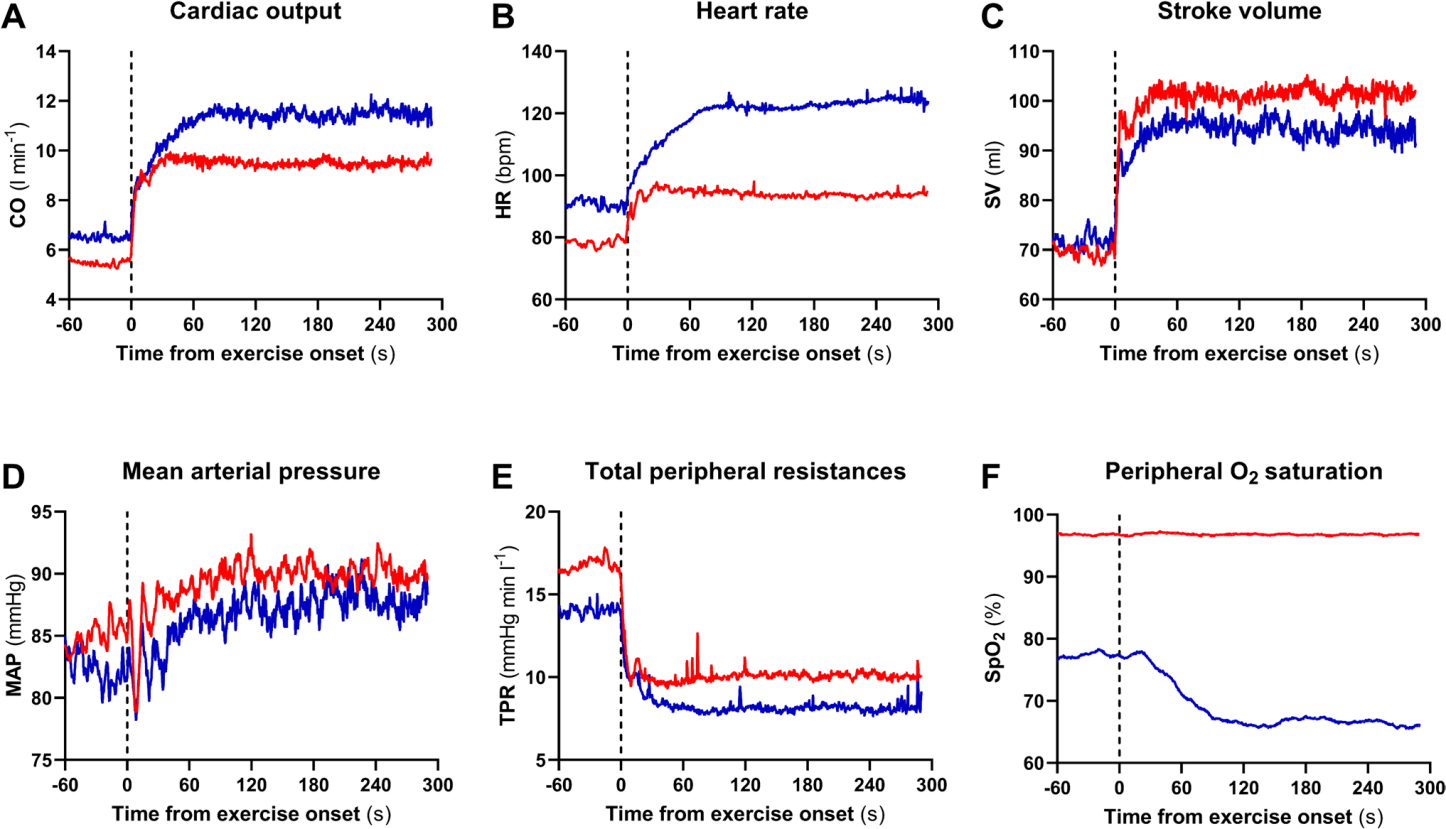
Time course of the cardiac output (CO), heart rate (HR), stroke volume (SV), mean arterial pressure (MAP), total peripheral resistances (TPR), and peripheral blood oxygen saturation (SpO_2_) during the rest to 50 W exercise transient in normoxia (red line) and in hypoxia (blue line). Mean values from all subjects (n = 10). The time scale refers to the time elapsed from the exercise onset

**Fig. 2:**
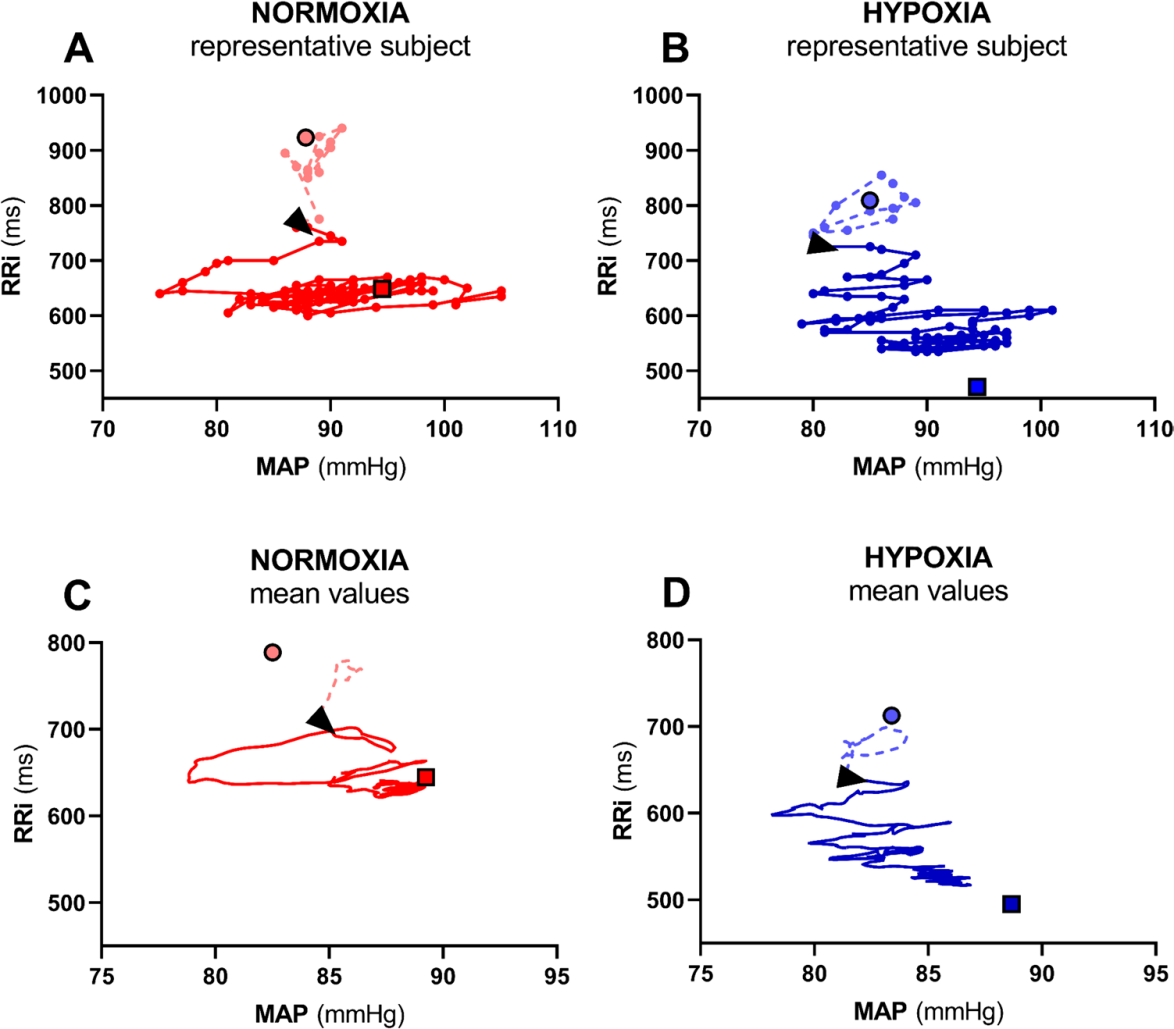
Contour plots of the relationship between R-to-R interval (RRi) and mean arterial pressure (MAP) from 10 s before to 60 s after exercise onset (black arrowhead). Panels A and B: beat-by-beat value from a representative subject with a time shift of 1 beat applied between MAP and RRi. Panels C and D: mean values obtained from all rest-to-exercise transients (n = 30) with a time shift of 1 beat applied between MAP and RRi. In all panels, dots and squares represent, respectively, rest and 50 W steady states

Data are presented as mean ± standard deviation. Two-way ANOVA for repeated measures was used to investigate differences in the four steady state conditions in order to isolate the effect of exercise and of hypoxia separately. One way ANOVA for repeated measures was used to investigate differences between MAP and BRS measured at different time points during exercise transients. Tukey’s multiple comparisons test was used to isolate differences when necessary. Student’s T test for repeated measures was used to compare Eq. 1 parameters obtained in N and H. Differences were considered significant when *p* < 0.05. The statistical software Prism (version 8, GraphPad®, La Jolla, CA, USA) was used. Data fitting with Eq. 1 was performed after superimposition of the three exercise transients for each subject in order to avoid timeline distortion due to averaging (Francescato et al. 2014a, b; Bringard et al. 2014); MATLAB (version 9.5.0.944444 with Curve Fitting Toolbox, The MathWorks, Inc., Natick, MA, USA) was used with this aim. Figure 1, all Panels and Fig. 2, Panels C-D report average data from all rest-to-exercise transients and from all subjects (n = 30) interpolated at 0.1 s.

## Results

Mean steady state data are reported in Table 1. All cardiovascular data changed from rest to exercise except DAP (both in N and H). Most data differed between H and N except for resting and exercising MAP, DAP, and [HCO_3_^−^], and resting SV, CO, and [La].

**Table 1:** Steady state values (mean ± SD) in the four experimental conditions

	Normoxia		Hypoxia	
	Rest	50 W	Rest	50 W
SAP (mmHg)	120 ± 9	145 ± 12 ^####^	127 ± 18**	164 ± 20****^, ####^
DAP (mmHg)	68 ± 13	68 ± 10	69 ± 11	68 ± 14
MAP (mmHg)	83 ± 12	89 ± 9 ^#^	83 ± 12	89 ± 14 ^#^
HR (min^−1^)	77 ± 9	94 ± 8 ^####^	86 ± 9**	123 ± 15****^, ####^
RRi (ms)	788 ± 90	645 ± 62 ^####^	713 ± 71***	494 ± 71****^, ####^
SV (ml)	75 ± 20	105 ± 24 ^####^	74 ± 17	96 ± 18*^,####^
CO (l min^−1^)	5.9 ± 1.9	9.9 ± 2.4 ^####^	6.3 ± 1.7	11.7 ± 2.1****^, ####^
TPR (mmHg min l^−1^)	15.8 ± 5.8	9.7 ± 2.9 ^####^	14.5 ± 5.3*	8.0 ± 2.5**^, ####^
BRS (ms mmHg^−1^)	14.7 ± 3.6	7.0 ± 3.0 ^####^	10.6 ± 2.8**	2.9 ± 1.5**^, ####^
SpO_2_ (%)	96 ± 4	97 ± 1	82 ± 5**	67 ± 6***^, ##^
[La] (mmol l^−1^)	1.26 ± 0.33	1.29 ± 0.75	1.06 ± 0.36	2.29 ± 1.16**^, ###^
pO_2_ (mmHg)	89 ± 11	91 ± 14	48 ± 8****	39 ± 6****^, ###^
pCO_2_ (mmHg)	39 ± 4	39 ± 5	33 ± 4*	30 ± 9**
pH	7.41 ± 0.01	7.43 ± 0.03	7.49 ± 0.04***	7.49 ± 0.05**
[HCO_3_^−^] (mmol l^−1^)	24.9 ± 1.3	24.7 ± 1.8	25.8 ± 1.5	25.7 ± 2.1

*SAP* systolic arterial pressure, *DAP* diastolic arterial pressure, *MAP* mean arterial pressure, *HR* heart rate, *RRi* R-to-R interval, *SV* stroke volume, *CO* cardiac output, *TPR* total peripheral resistances, *BRS* baroreflex sensitivity, *SpO*_2_ peripheral blood oxygen saturation, [*La*] blood lactate concentration, *pO*_2_ capillary blood partial pressure of oxygen, *pCO*_2_ capillary blood partial pressure of carbon dioxide, [*HCO*_3_^−^] blood bicarbonate concentration, * statistically different from the corresponding value in normoxia (*: p < 0.05; **: p < 0.01; ****: p < 0.0001), ^#^ statistically different from the corresponding value at rest (^#^: p < 0.05; ^##^: p < 0.01; ^###^: p < 0.001; ^####^: p < 0.0001)

The time course of the main investigated parameters is shown in Fig. 1. In H, with respect to N, the exercise transient showed a greater increase of CO and HR, a slightly lower SV, MAP and TPR, and a progressive decrease of SpO_2_.

During the rest-to-exercise transient in N, MAP showed a sudden decrease at the very beginning of exercise (Fig. 1, Panel D, red line). Minimum MAP was 73 ± 11 mmHg (p = 0.0002 and p < 0.0001 vs. rest and 50 W steady state, respectively) and appeared after 8.1 ± 1.8 s from exercise onset. Such a pattern was not observed during the same transient in H (Fig. 1, Panel D, blue line). This implied that the pattern of the MAP-RRi relationship differed between conditions, as shown by Fig. 2﻿.

**Fig. 3:**
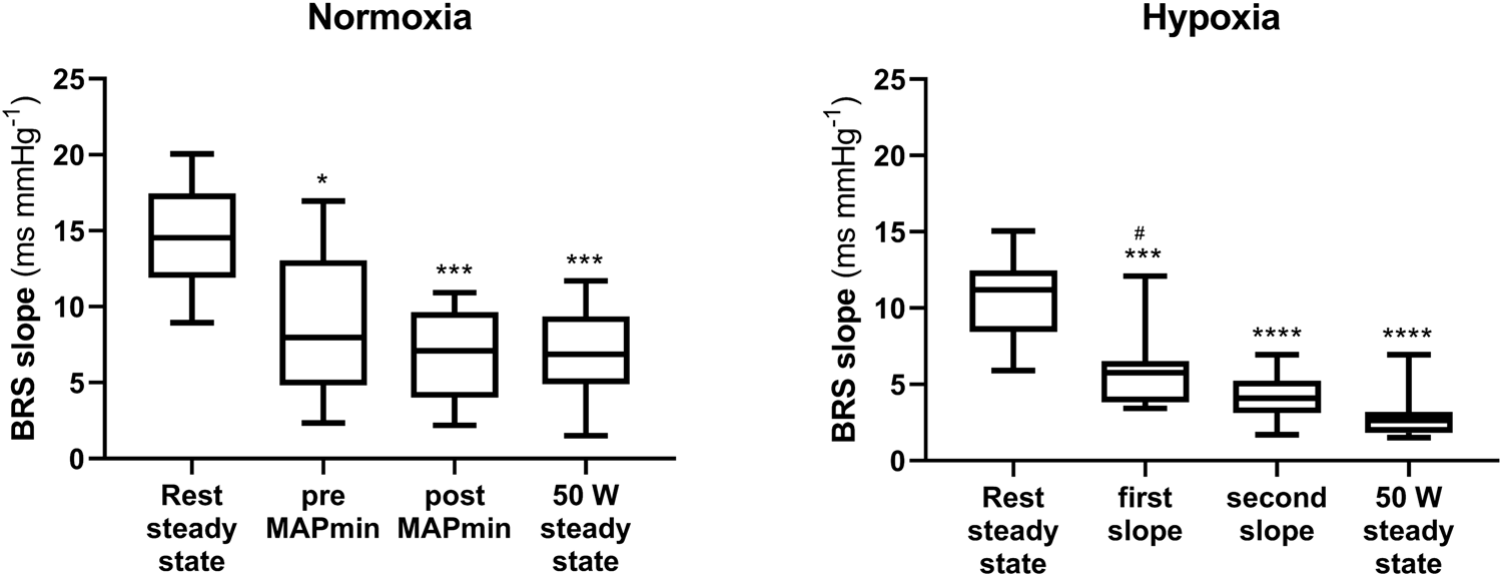
Tukey representation of the baroreflex sensitivity (BRS) measured at different time points in normoxia and in hypoxia. *: significantly different vs. rest steady state (**: p < 0.01; ***: p < 0.001; ****: p < 0.0001); ^#^: significantly different vs. exercise steady state (p < 0.05). MAPmin: minimum mean arterial pressure

The BRS measured during the exercise transients are reported in Fig. 3. In N, the first slope was always computed on sequences that occurred before minimum MAP was reached, and thus was characterised by consensual decrease in MAP and RRi. The BRS values before and after minimum MAP were lower than in resting steady state (p ≤ 0.0148), but similar to those during exercise steady state (p ≥ 0.6796). In H, the first BRS slope was taken regardless of its direction and 22 out of 29 sequences were characterised by consensual decrease in MAP and RRi. Moreover, these first sequences appeared within 3.6 ± 3.1 s after the exercise onset. As in N, they were lower than in resting steady state (p = 0.0001).

Parameters obtained from the analysis of the CO and HR kinetics are reported in Table 2. Both the CO and HR baselines were higher in H than in N. Regarding phase I, CO A_1_, CO τ_1_, and HR A_1_ were similar in H and N whereas HR τ_1_ was higher in H than in N. Regarding phase II, both CO and HR A_2_ and τ_2_ were higher in H than in N.

**Table 2:** Mean ± standard deviation and (95% confidence interval) of the parameters of the cardiac output (CO) and heart rate (HR) kinetics as obtained by data fitting with Eq. 1

			Phase I			Phase II	
		Baseline (l min^−1^ or min^−1^)	Amplitude (l min^−1^ or min^−1^)	Time constant (s)	Time delay (s)	Amplitude (l min^−1^ or min^−1^)	Time constant (s)
CO	N	5.46 ± 1.38	2.96 ± 1.51	2.1 ± 1.0	11.4 ± 13.3	1.13 ± 0.86	9.1 ± 4.4
		(4.47–6.45)	(1.87–4.04)	(1.3–2.8)	(0.3–22.5)	(0.52–1.75)	(5.4–12.8)
	H	6.58 ± 1.62***	2.31 ± 0.94	2.3 ± 1.8	12.8 ± 3.4	2.68 ± 0.78**	23.8 ± 15.4*
		(5.42–7.74)	(1.63–2.97)	(1.1–3.6)	(10.3–15.2)	(2.12–3.25)	(12.8–34.8)
HR	N	78 ± 9	7.7 ± 4.6	0.8 ± 1.0	5.6 ± 5.4	7.8 ± 5.5	4.0 ± 3.8
		(72–85)	(4.4–11.0)	(0.1–1.6)	(1.5–9.7)	(3.8–11.7)	(1.1–6.9)
	H	93 ± 12***	7.1 ± 5.9	2.9 ± 2.7*	18.9 ± 13.1	22.8 ± 8.2****	26.9 ± 13.3***
		(85–101)	(2.9–11.3)	(0.4–5.5)	(9.6–28.3)	(16.9–28.6)	(17.5–36.4)

Individual data are reported in the Supplementary material (Tables S1 and S2, respectively for CO and HR).

*N* normoxia, *H* hypoxia, * significantly different vs. corresponding N value (*: p < 0.05; **: p < 0.01; ***: p < 0.001; ****: p < 0.0001)

## Discussion

### Arterial baroreflex at steady state

At rest, BRS around the OP was lower in H than in N, in line with previous observations (Koller et al. 1988; Lucy et al. 2000; Buchheit et al. 2004; Siebenmann and Lundby 2015; Siebenmann et al. 2019). This may be a consequence of a lower vagal output to the heart. In fact, BRS is reduced by full vagal blockade with atropine (Fontolliet et al. 2018), and enhanced in conditions characterised by a strong vagal output to the heart, such as supine posture (O’Leary et al. 2003; Steinback et al. 2005), water immersion (Chouchou et al. 2020), and short-term microgravity exposure (Hirayanagi et al. 2004; Di Rienzo et al. 2008). Moreover, the decrease of vagal activity in H is often associated with an increased sympathetic activity (Robinson et al. 1966; Saito et al. 1988; Hopkins et al. 2003; Ferretti et al. 2005; Tan et al. 2013; Siebenmann et al. 2015, 2019).

The lower BRS in H was coupled with a displacement of the OP toward lower RRi without any changes in MAP (Table 1), indicating resetting of the baroreflex in H. It was previously suggested that a hypoxic hyperventilation may be responsible for a decrease in the arterial baroreflex gain (Melcher 1980; Mancia and Mark 1983). In agreement with this hypothesis, the maximum baroreflex gain, obtained with the neck pressure-suction technique, was found to be lower than at sea level at altitudes above 4000 m (Sagawa et al. 1997). Since a F_I_O_2_ of 0.11 corresponds to a simulated altitude around 5000 m, the observed BRS decrease in the present study may in fact reflect a reduction of the maximum gain of the open-loop arterial baroreflex relationship. However, the observed decrease of the BRS around the OP in H may also be due to an OP displacement along the baroreflex curve away from the point of maximum gain, *i.e.* the centring point, as occurs during exercise (Potts et al. 1993; Norton et al. 1999; Fadel et al. 2001; Ogoh et al. 2003, 2005; Raven et al. 2006). To the best of our knowledge, no open-loop based studies of the arterial baroreflex have analysed the relative OP position in acute hypoxia.

At exercise, the BRS measured around the OP was lower in H than in N due to a BRS decrease of similar extent from rest to exercise steady states (−7.4 ± 4.6 ms mmHg^−1^ in N and −8.0 ± 3.4 ms mmHg^−1^ in H, p = 0.6158). This observation is compatible with residual vagal activity in resting H. If this is so, BRS decrease during the exercise transient might be the result of an additional vagal withdrawal, not only in N but also in H. In this case, the reduction of resting vagal activity in H would be partial. We can speculate that it may be greater the stronger the level of acute hypoxia to which a subject is exposed.

### Baroreflex dynamics during the exercise transient

In N, MAP and TPR showed a sudden fall at exercise onset (Fig. 1, Panel D and E), in line with previous findings (Bringard et al. 2017), possibly due to prompt muscle vasodilation (Rådegran and Saltin 1998). The observed MAP fall was coupled with a RRi decrease as in a baroreflex relationship, so that BRS could be measured, as previously proposed (Bringard et al. 2017; Taboni et al. 2021a, b). The measured BRS of this baroreflex sequence was significantly lower than that measured at rest, and similar to that at exercise (Fig. 3), thus witnessing a prompt decrease of the BRS around the OP, similarly to previous observations (Bringard et al. 2017). When passing from rest to exercise, the open-loop arterial baroreflex relationship is displaced without any changes in maximal gain but with a shift of OP away from the centring point (Potts et al. 1993; Norton et al. 1999; Fadel et al. 2001; Ogoh et al. 2003, 2005; Raven et al. 2006), so that the BRS decrease measured upon exercise onset may simply represent this OP shift. The BRS decrease around OP occurs rapidly, within the very first few seconds of exercise, therefore only a very fast mechanism may be implied. It has been hypothesised that this mechanism may be the sudden withdrawal of the vagal tone, since the activation of the sympathetic system would require a longer time (Warner and Cox 1962; Fagraeus and Linnarsson 1976; Lador et al. 2006, 2013; Fontolliet et al. 2021).

In H, contrary to N, MAP did not show a single transient nadir but rather multiple oscillatory adjustments (Fig. 1, Panel D and Fig. 2), whereas TPR showed a similar initial fall upon exercise onset in the two conditions despite a lower baseline value in H (Fig. 1, Panel D). This apparently different MAP control at exercise onset in H may be due to a greater baseline sympathetic drive than in N (Saito et al. 1988; Rowell et al. 1989; Halliwill and Minson 2002), which would sustain arterial blood pressure and counteract hypoxic vasodilation (Weisbrod et al. 2001; Halliwill and Minson 2002). In H, several oscillations were observed before the attainment of a new steady state MAP value (Fig. 1, Panel D and Fig. 2) and during these MAP oscillations, it was possible to identify several baroreflex sequences. The BRS around OP promptly decreased since the first sequence after exercise start (Fig. 3), similarly to N. This suggests that the mechanism leading to the BRS decrease may be the same in the two analysed conditions, so that vagal withdrawal can still partly determine it. This would reinforce the concept that some level of vagal activity is still present in H.

Concerning baroreflex resetting during the exercise transient, in N the results are in line with previous literature (Bringard et al. 2017; Fagoni et al. 2020). The attainment of minimum MAP may trigger the resetting process. In H instead, it is hard to identify clear patterns of resetting. Although the mean data in H suggest the attainment of a minimum MAP similar to that in N (Fig. 2, panel D), this is not easily identifiable in all subjects. The progressive decrease of SpO2 during the exercise transient in H suggests that other factors than baroreflex mechanisms or stronger sympathetic stimulation may participate in the HR response to exercise onset (Halliwill et al. 2003), which make the patterns followed by HR and MAP more complex than in N.

### Cardiovascular dynamics during the exercise transient

When analysing the rest-to-exercise transient, the phase I of the CO kinetics showed no significant differences in both amplitude and time constant in the two conditions (Table 2). This result goes against previous literature. In fact, when analysing a rest-to-50 W exercise transient in the same hypoxic conditions, lower A_1_ and lower τ_1_ were found in H than in N (Lador et al. 2008). This discrepancy may be due, at least in part, to the fact that in this study the number of participants was twice that of Lador et al (2008). Notwithstanding this, we note that, during full parasympathetic blockade, the CO kinetics at exercise onset shows a clear phase I, though with a smaller A_1_ than in control (Fontolliet et al. 2021). This was ascribed to sudden increase of SV by vagus-independent mechanisms active upon exercise onset, such as an increase of pre-load via muscle pump action (Chung et al. 1997; Sundblad et al. 2000, 2014; Naeije and Badagliacca 2017; Fagoni et al. 2020) and a reduction of after-load via prompt vasodilation at the level of the contracting muscles (Ferretti et al. 1995; DeLorey et al. 2003; Clifford 2007; Chin et al. 2010). Moreover, the phase I of the HR kinetics was characterised by similar amplitude and higher time constant in H than in N (Table 2). This is, at odds with the same hypoxic transient of Lador et al (2008), where HR A_1_ was lower and HR τ_1_ was equal compared to N. The phase I of HR responses to exercise onset is abolished under full parasympathetic blockade with atropine (Fontolliet et al. 2021). We speculate that, in H, the A_1_ of CO and HR were partially affected by vagal withdrawal, in line with the previous observation that at rest some degree of vagal activity may subsist. Notably, cardiovascular responses in N and H might have been influenced also by the respiratory apparatus used in the two conditions. In fact, in H a low resistance, two-way non-rebreathing T-shape valve was mounted at the mouth of each participant which increased the dead space by approximately 50%.

The phase II of both CO and HR was characterised by higher A_2_ and τ_2_ in H than in N, in line with previous findings (Lador et al. 2013). The current interpretation is that the incurring sympathetic stimulation may play a major role during this phase (Lador et al. 2006, 2013; Fontolliet et al. 2021), triggered by a more intense muscle-metabolic reflex (Houssiere et al. 2005) and chemoreflex (Jouett et al. 2015; Keir et al. 2019), given the progressive further SpO_2_ decrease after the exercise onset (Fig. 1, Panel F). Of course, a higher τ_2_ of CO should carry along a higher τ_2_ of oxygen uptake, which was not analysed in this study. Yet a hint suggesting that this may indeed be the case comes from the steady state lactate values in H, indicating early lactate accumulation in the exercise transient (Ferretti et al. 2022) leading to higher lactate steady state at exercise than at rest.

## Conclusions

Upon exercise onset in acute normobaric hypoxia, the immediate fall of peripheral resistances at exercise start was not accompanied by a dramatic fall of mean arterial pressure. This last showed several oscillations until reaching a new steady state value. Moreover, the baroreflex sensitivity decreased immediately and was associated with the presence of a phase I of heart rate responses. These fast cardiovascular readjustments upon exercise onset are compatible with a withdrawal of residual vagal activity concomitant with increased sympathetic drive. After the first cardiovascular adjustments, the attainment of the new steady state was slower in hypoxia than in normoxia, as long as acute hypoxia was characterised by further peripheral deoxygenation.

### Supplementary Information

Below is the link to the electronic supplementary material.

Supplementary file1 (XLSX 12 KB)

Supplementary file2 (XLSX 12 KB)

## Data Availability

The datasets generated and analysed during the current study are available from the corresponding author on reasonable request.

## Abbreviations

[HCO_3_^−^]: Bicarbonate concentration
[La]: Blood lactate concentration
A: Amplitude of the bi-exponential model, subscripts 1 and 2 refers to phase I and phase II, respectively
*b*: Baseline value of the bi-exponential model
BRS: Spontaneous baroreflex sensitivity
CO: Cardiac output
*d*: Time delay of the bi-exponential model
DAP: Diastolic arterial pressure
H: Hypoxic condition
HR: Heart rate
MAP: Mean arterial pressure
N: Normoxic condition
OP: Operating point
pCO_2_: Carbon dioxide partial pressures
pO_2_: Oxygen partial pressures
RRi: R-to-R interval
SAP: Systolic arterial pressure
SpO_2_: Peripheral blood oxygen saturation
SV: Stroke volume
TPR: Total peripheral resistances
τ: Time constant of the bi-exponential model, subscripts 1 and 2 refers to phase I and phase II, respectively
